# *Dispec:* A Novel Peptide Scoring Algorithm Based on Peptide Matching Discriminability

**DOI:** 10.1371/journal.pone.0062724

**Published:** 2013-05-13

**Authors:** Chuan-Le Xiao, Xiao-Zhou Chen, Yang-Li Du, Zhe-Fu Li, Li Wei, Gong Zhang, Qing-Yu He

**Affiliations:** 1 Key Laboratory of Functional Protein Research of Guangdong Higher Education Institutes, Institute of Life and Health Engineering, College of Life Science and Technology, Jinan University, Guangzhou, China; 2 School of Mathematics and Computer Science, Yunnan University of Nationalities, Kunming, China; 3 Jinan University Network and Educational Technology Center, Guangzhou, China; UGent/VIB, Belgium

## Abstract

Identifying peptides from the fragmentation spectra is a fundamental step in mass spectrometry (MS) data processing. The significance (discriminability) of every peak varies, providing additional information for potentially enhancing the identification sensitivity and the correct match rate. However this important information was not considered in previous algorithms. Here we presented a novel method based on Peptide Matching Discriminability (PMD), in which the PMD information of every peak reflects the discriminability of candidate peptides. In addition, we developed a novel peptide scoring algorithm Dispec based on PMD, by taking three aspects of discriminability into consideration: PMD, intensity discriminability and *m/z* error discriminability. Compared with Mascot and Sequest, Dispec identified remarkably more peptides from three experimental datasets with the same confidence at 1% PSM-level FDR. Dispec is also robust and versatile for various datasets obtained on different instruments. The concept of discriminability enhances the peptide identification and thus may contribute largely to the proteome studies. As an open-source program, Dispec is freely available at http://bioinformatics.jnu.edu.cn/software/dispec/.

## Introduction

In the analysis of mass spectrometry (MS), the mass of each peptide is measured and then selected and fragmented to obtain MS/MS spectra [1]. These second-order spectra are identified by algorithms to determine peptide sequences. Large amount of spectra obtained from LC-MS/MS experiments sets a challenge to the identification of peptides [2], [3]. A number of peptide identification algorithms for MS data analysis are available, and each of them uses different ways to select significant peaks, compare the peaks to the theoretical peaks and score the similarity [4]–[11]. However, a type of important information from the spectra, the discriminability of each peak, was not considered in any of these algorithms [4]–[13].

Discriminability of a peak (MS/MS fragmentation peak) is a type of score that characterizes the confidence of peptide matching: distinguishability of the matched peptide from other peptides, and the distinguishability of the real fragment ion from random ones. It comprises of three aspects: peptide matching discriminability for candidate peptides, intensity discriminability and *m/z* error discriminability between theoretical and experimental spectra (details see below). The peptide matching discriminability of each peak for candidate peptides can differ largely, providing various quality and confidence information. It is a property of the peak itself, not derived from any overall statistics of the spectra, can thus serve as additional independent information to improve the sensitivity and the confidence of the identification. We developed a novel model of Peptide Matching Discriminability (PMD) to calculate the discriminability of each peak for candidate peptides from MS/MS spectra. We further developed an open-source program Dispec based on the PMD model and performed a comparison test with other algorithms using the standard 18 proteins dataset and *E. coli* proteome dataset. Dispec demonstrated higher sensitivity and confidence in identifying peptides from different MS datasets at 1% PSM-level false discovery rate (PSM-level FDR), implicating that the PMD concept provides important insight for peptide identification.

## Methods

### Mass Spectrometry Datasets and Data Preprocessing

A dataset of 18 standard proteins mixture was used to test the accuracy, robustness and versatility of Dispec. The dataset measured by four instruments (Thermo Finnigan LTQ-FT, Thermo Finnigan LCQ DECA, Thermo Finnigan LTQ and Micromass/Waters QTOF Ultima, abbreviated below as FT, LCQ, LTQ and QTOF, respectively) was obtained from https://regis-web.systemsbiology.net//PublicDatasets/ [14]. This dataset is widely used to validate peptide scoring algorithms and to test the dynamic range of the algorithm [15]. The LTQ-Orbitrap data obtained from the *S. pneumoniae* D39 protein identification (http://bioinformatics.jnu.edu.cn/software/dispec/) containing more than 270,000 spectra served as training dataset for parameters of the model [16]. The dataset of *E. coli* proteome was obtained from http://marcottelab.org/MSdata/Data_03/ [17].

For *S. pneumoniae* D39 and *E. coli* datasets, the raw format files were converted to dta file format by Bioworks 3.31 (Thermo Finnigan, San Jose, CA). For the 18 proteins dataset, the dta format files were obtained from the website. All the dta format files were merged to Mascot generic format (mgf) by the merge.pl program (http://www.matrixscience.com/downloads/merge.zip). The dta format files were the input files of Dispec and Sequest software.

### MS/MS Database Search

For target-decoy based FDR calculation, the D39 database contains 1914 real protein sequences and the built forward/reverse database contains 3828 protein sequences; the 18 proteins database contains 1822 real protein sequences and the built forward/reverse database contains 3644 protein sequences; the *E. coli* database contains 4279 real protein sequences and the built forward/reverse database contains 8558 protein sequences. Mascot 2.3 search engine (Matrix Science, London, UK) was used to search the Mascot generic format (mgf) files. The dta format files were searched using the Sequest search engine (Thermo Fisher Scientific, Waltham, MA, version 28.13) and Dispec. For Mascot, Sequest and Dispec, the following search criteria were applied: full tryptic specificity was required; two missed cleavages were allowed; Cys (+57.021464 Da, Carbamidomethylation) was set as fixed modification, whereas Met (+15.994915 Da, Oxidation) was considered as variable modifications.

The precursor ion mass tolerances and fragment ion mass tolerances vary according to the instrument type (Table 1). The fragment ion tolerance of Sequest was set to 1.0 Da since it requires integer value for *m/z* [4].

**Table 1:** The parameters of precursor and fragment ion tolerance according to instrument type.

Instrument Type	Dispec and Mascot		Sequest	
	Precursor ion tolerance	Fragment ion tolerance	Precursor ion tolerance	Fragment ion tolerance
LCQ_Deca	3.0 Da	0.5 Da	3.0 Da	1.0 Da
LTQ	3.0 Da	0.5 Da	3.0 Da	1.0 Da
LTQ-FT	10 ppm	0.5 Da	10 ppm	1.0 Da
QTOF	0.2 Da	0.2 Da	10 ppm	1.0 Da
LTQ-Orbitrap	10 ppm	0.5 Da	10 ppm	1.0 Da

### False Discovery Rate (FDR) at PSM-level

The peptide spectrum matches (PSMs) with the top rank were extracted from the Mascot data file (.dat) with our in-house Matlab program and exported to calculate FDR threshold at PSM-level. PSMs of Sequest results with the top rank and Δ*Cn* ≥0.1 were extracted from Sequest output files (.out) and exported to calculate FDR threshold at PSM-level. Dispec results and the extracted results of Mascot and Sequest were written to csv format files. All target and decoy scores with the best ranking PSMs were sorted in ascending order to calculate its FDR at PSM-level value by Kall's method [18]–[20]. FDR at PSM-level was calculated as the ratio between the number of decoy and target PSMs above threshold.

The scoring functions vary in different search algorithms. For Mascot, the ion scores were sorted to calculate FDR at PSM-level when peptide length ≥6; for Sequest, the *Xcorr* scores were sorted to calculate FDR at PSM-level by different precursor charges when peptide length ≥6 and Δ*Cn* ≥0.1; for Dispec, the *Sp* scores were sorted to calculate FDR at PSM-level when peptide length ≥6.

All the score thresholds of 1% FDR at PSM-level were calculated by our Matlab program. The number of identified unique peptides was compared at FDR≤0.01.

### Training Dataset of Intensity and *m/z* Error Discriminability

The identification result of D39 dataset at PSM-level FDR≤0.01, including 97535 spectra and 3570 unique peptides, was considered as high-confidence and correct result. The corresponding reversed sequences of these peptides were considered as incorrect peptides. These high-confidence peptides and incorrect peptides serve as the training set of statistical analysis.

## Results and Discussion

### Peak Selection

In the Dispec algorithm, peaks closer than 1±0.25 Da are considered as isotope peaks and were filtered [9], [10]. The range between maximum and minimum *m/z* values of the experimental spectrum was divided into 10 equal bins. The 20 most intense peaks in every bin were selected and the intensity of each selected peak was normalized against the highest intensity [4], [11].

### Theoretical Spectra

The theoretical spectra were generated according to the scenario of peptide bonds’ breakage. We considered b/y fragment ions and a loss of b-H_2_O or y-H_2_O when the b, y fragment ions contain S, T, E, D amino acids; or a loss of b-NH_3_ or y-NH_3_ if the b, y fragment ions contain R, K, Q, N amino acids. For parent ions with charge ≥ +1, we considered +1 charge fragment ion peaks. For parent ions with charge ≥ +2 and their fragment ions contain one of the R, K, H amino acids, we considered +2 charge fragment ion peaks [5], [9], [10], [21].

### Peptide Matching Discriminability (PMD) for Candidate Peptides

A selected experimental peak in the MS/MS spectra matches one peptide if it matches at least one theoretical fragment ion peak of this peptide. The peptide matching number of candidate peptide of each selected peak was calculated as *M_i_* (*i = *1, 2, …, n). We then calculated the average peptide matching number of all matched peak:
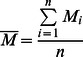



*M_i_* = the peptide matching number of candidate peptide.

*n* = the peak number of matched candidate peptide.


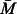
 *= *the average peptide matching number of all matched peak.

The peptide matching discriminability 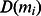 of each peak can be then calculated as
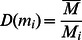

*D(m_i_) = *peptide matching discriminability of the *i*-th peak. Compared with other peaks of this MS/MS spectrum, it reflects the peptide matching confidence of this peak.

**Figure 1:**
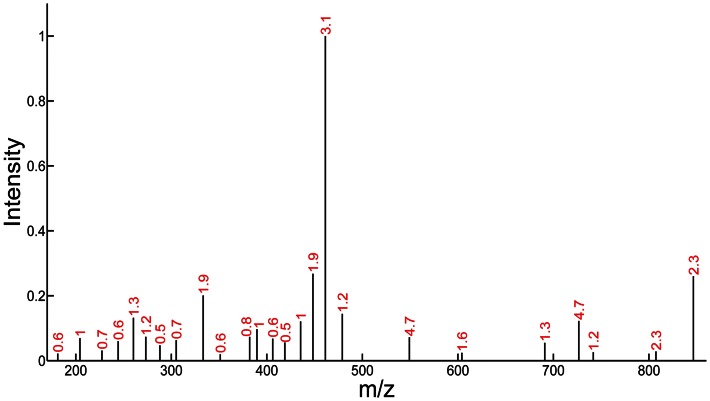
Peptide Matching Discriminability (PMD) for the selected peaks (black bars). The peptide matching discriminability values for peaks are marked as red numbers.

The selected peaks and the peptide matching number of candidate peptides were shown as PMD for candidate peptides (Figure 1, Figure S1). Importantly, the peaks with higher intensities do not necessarily possess higher discriminability.

### Statistical Analysis for Intensity Discriminability

In many previous algorithms the intensity information was used to calculate the similarity score between the experimental peaks and the theoretical peaks [4], [8], [10], [11], [22]. For consistency with PMD information, we defined intensity discriminability to utilize the peak intensity information. We divided the normalized peak intensity range [0, 1] into 10 equal intervals with an additional category of the highest peak for rounding convenience: [0, 0.1), [0.1, 0.2), [0.2, 0.3), …, [0.9, 1.0) and 1.0. The correct and random matching number in the training dataset (*S. pneumoniae* D39 dataset) can be statistically calculated in each interval. In the *j*-th (*j = *1, 2, …,11) interval, the intensity discriminability 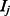 is calculated as 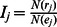, where 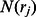
is the correct matching number and 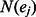 is the random matching number. The intensity discriminability of b-ions 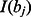, y-ions 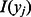 and the six types (b, b-H_2_O, b-NH_3_, y, y-H_2_O and y-NH_3_) of theoretical ions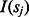 were statistically obtained from the training dataset, as listed in Table 2.

**Table 2:** The intensity discriminability of b-ions (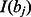), y-ions (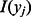) and the six types (b, b-H_2_O, b-NH_3_, y, y-H_2_O and y-NH_3_) of theoretical ions (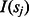).

Range	[0, 0.1)	[0.1, 0.2)	[0.2, 0.3)	[0.3, 0.4)	[0.4, 0.5)	[0.5, 0.6)	[0.6, 0.7)	[0.7, 0.8)	[0.8, 0.9)	[0.9, 1.0)	1.0
*I*(*b_j_*)	1.14	2.61	4.03	5.29	5.95	6.21	6.98	6.63	5.24	4.69	3.67
*I*(*y_j_*)	4.03	13.39	23.69	30.40	29.95	26.05	26.32	31.62	37.41	48.42	35.63
*I*(*s_j_*)	1.48	1.96	2.05	2.21	2.34	2.39	2.26	2.31	2.43	2.57	3.09

### Statistical Analysis for *m/z* Error Discriminability

In some algorithms, e.g. pNovo, MassWiz and DeltAMT [7], [15], [21], the *m/z* error between the experimental peaks and the theoretical peaks was considered when calculating similarity score. Some studies [15], [23] showed that the *m/z* error distribution remarkably differs between correct and wrong peptide match peaks. The *m/z* error of correct match peaks was mainly less than 1/5 of the error window, whereas the *m/z* error of wrong match peaks can be as high as the rest window [23]. This difference provides independent and additional information reflecting the correct match probability. Therefore, we introduced *m/z* error discriminability in our algorithm.

Similar to the intensity discriminability, the *m/z* error interval [0, 0.5] between experimental and theoretical fragment ions was divided into 10 equal intervals and an additional category of 0.5: [0, 0.05), [0.05, 0.1), [0.1, 0.15), …, [0.45, 0.5), 0.5. The correct and random matching number in the training dataset (*S. pneumoniae* D39 dataset) can be statistically calculated in each interval. In the *j*-th (*j = *1, 2, …, 10) interval, the *m/z* error discriminability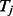 was calculated by the formula 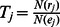, where 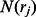
is the correct matching number and 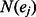 is the random matching number. The m/z error discriminability of b-ions 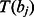, y-ions 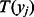
and the six types (b, b-H_2_O, b-NH_3_, y, y-H_2_O and y-NH_3_) of theoretical ions 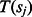 were statistically obtained from the training dataset, as listed in Table 3.

**Table 3:** The *m/z* error discriminability of b-ions (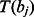), y-ions (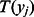) and the six types (b, b-H_2_O, b-NH_3_, y, y-H_2_O and y-NH_3_) of theoretical ions (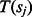).

Range	[0, 0.05)	[0.05, 0.1)	[0.1, 0.15)	[0.15, 0.2)	[0.2, 0.25)	[0.25, 0.3)	[0.3, 0.35)	[0.35, 0.4)	[0.4, 0.45)	[0.45, 0.5)
*T*(*b_j_*)	2.14	1.96	1.74	1.53	1.29	1.00	0.76	0.62	0.55	0.54
*T*(*y_j_*)	11.17	9.32	6.92	5.22	4.65	4.22	3.25	2.69	2.37	2.25
*T*(*s_j_*)	1.99	1.88	1.70	1.56	1.49	1.43	1.31	1.23	1.17	1.13

### Scoring Function

The scoring process of Dispec algorithm utilizes the above three types of discriminability information to evaluate the identification and matches. The scoring model based on PMD mainly considers three aspects: fragment ion matches, consecutive fragment ion matches and b/y fragment ion matches [21]. Each candidate peptides are scored and the scoring function is described as follows.

#### Fragment ion scoring

When matching an experimental peak to theoretical peak of fragment ion from a peptide in fragment error tolerance, the fragment ion discriminability of the *j*-th matching peak is defined as 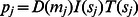. The total discriminability is 
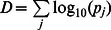
 which is equivalent to 
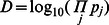
, and the discriminability score of all matching ions is 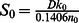, where

*k_0_* = number of matched peaks in the experimental spectrum.

*n_0_* = number of theoretical fragment peaks.

0.1406 = random matching probability of theoretical spectrum, which reflects the matched ability between experimental spectrum and decoy theoretical spectrum and is calculated from the training dataset using the following formula:



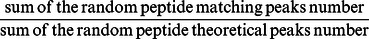



#### Consecutive fragment ion scoring

Multiple consecutive ion matches can be converted into a series of ion pairs matches: *N* multiple consecutive ions matches are converted into *N*-1 two consecutive ion matches, for example, if b_1_, b_2_ and b_3_ ions are consecutively matched, this consecutive ion match is converted into two match pairs: b_1_–b_2_ and b_2_–b_3_. The total discriminability of consecutive matches is 
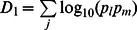
 and the score of consecutive matches is 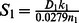, where

*p_l_* = the discriminability of the *l-*th matched peak.

*p_m_* = the discriminability of the *m*-th matched peak. Here, a consecutive ion match comprises of the *l* and *m* matches.

*k_1_* = number of consecutive matches in the experimental spectrum.

*n*_1_ = number of theoretical consecutive matches.

*0.0279 = *random consecutive matching probability of theoretical spectrum, which reflects the consecutive matching ability between experimental spectrum and decoy theoretical spectrum and is calculated from the training dataset by the following formula:



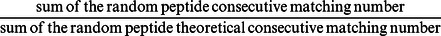



#### b/y-fragment ion scoring

The intensity and *m/z* error discriminability of b/y-ions (especially for y-ion) are mostly more than the discriminability of the six ion types (Table 2 and Table 3). This implies that b/y-ions matches are more efficient in the identification. Hence, the b/y-ion discriminability is separately considered in the scoring function. To score the b/y-ion peaks, the b/y-ion discriminability is firstly calculated as:
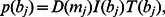


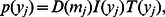



Or 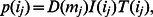 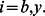


And the score of b/y-ion match is then calculated:
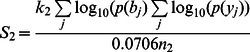



where

*k_2_* = number of the peaks matching to b-ions and y-ions

*n_2_* = number of b-ions and y-ions producing by theoretical spectra

0.0706 = b/y-ions random matching probability of theoretical spectrum, which reflects the b/y-ions matching ability between experimental spectrum and decoy theoretical spectrum and is calculated from the training dataset by the following formula:



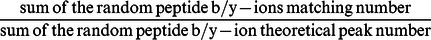



The overall score 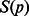 is the sum of the above three scores: 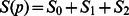.

### Comparison of Dispec with Mascot and Sequest

We compared our algorithm Dispec (Matlab version) with two widely-used MS identification algorithms Mascot and Sequest using three datasets: in-house generated *S. pneumoniae* D39 dataset, 18 standard proteins mixture and *E. coli* datasets.

**Figure 2:**
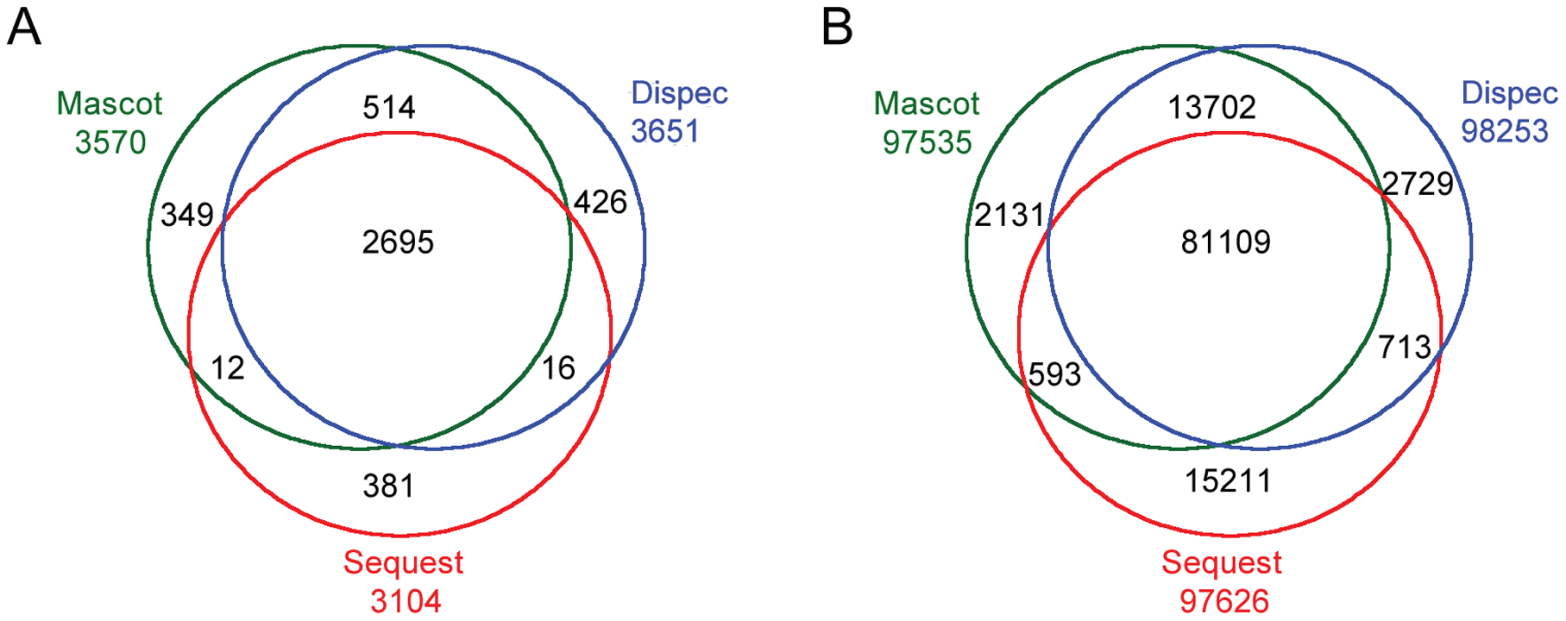
Comparison of Mascot, Sequest and Dispec using *S.* pneumoniae D39 dataset. (A) Number of identified peptides. (B) Number of identified spectra.

**Figure 3:**
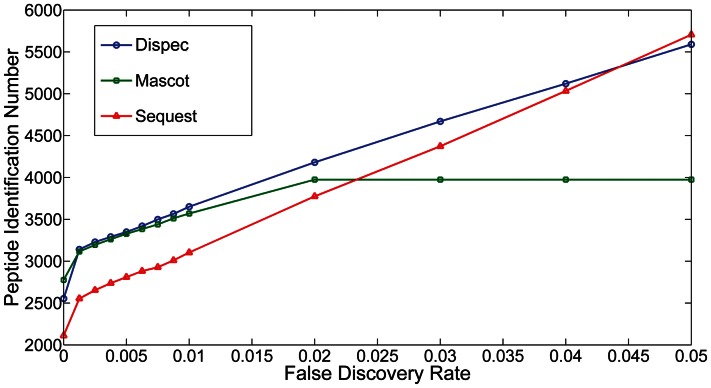
The identified peptide number versus PSM-level false discovery rate (PSM-level FDR) for Dispec, Mascot and Sequest within the PSM-level FDR range 0%∼5%.

In terms of the *S. pneumonia* D39 dataset, all algorithms were able to identify more than 3000 peptides and more than 97500 spectra under the criteria PSM-level FDR ≤0.01 (Figures 2A and 2B). Most of the peptides (2695) and spectra (81109) could be identified by all the three algorithms. The overlap ratio of identified peptides and spectra from Mascot and Dispec are as high as 89.9% and 97.2%, showing a good consistency of Dispec with other algorithms. As shown in Figure 3, Dispec identified more peptides and spectra than Mascot and Sequest in the PSM-level FDR range of 0.2%∼4% [18].

**Figure 4:**
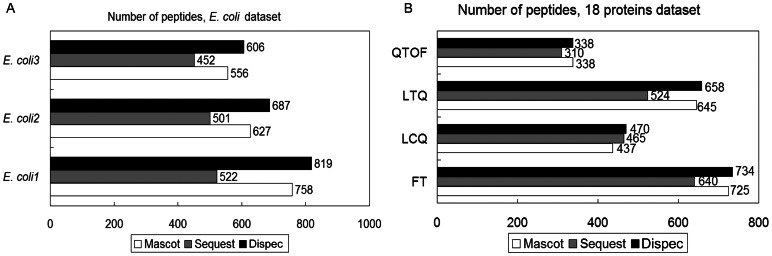
The number of identified peptides at 1% PSM-level FDR from *E.* coli dataset (A) and the standard 18 proteins dataset (B) using Dispec, Mascot and Sequest.

In terms of the publicly available standard 18 proteins dataset obtained using four types of MS instruments (FT, LTQ, LCQ and QTOF) and *E. coli* dataset (LTQ-Orbitrap), we tested Dispec’s adaptability under PSM-level FDR ≤0.01 (Figure 4). Compared with Mascot and Sequest, Dispec identified more peptides than Mascot in all MS data, showing its robust power of identification, stability and extensiveness.

### The Number of High-confidence Peptides Identified

**Figure 5:**
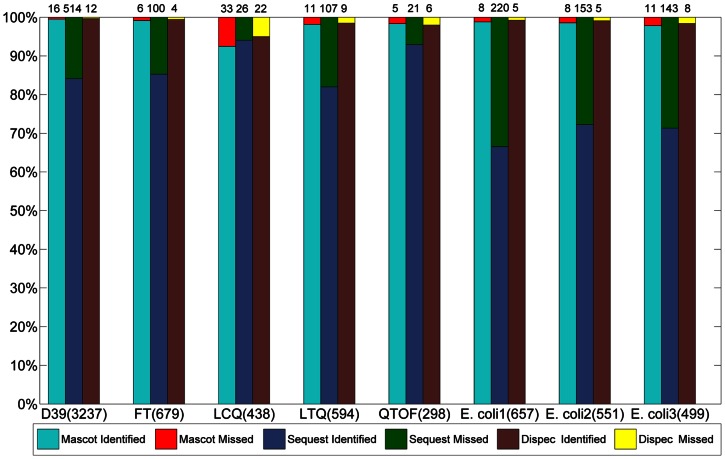
The high-confidence peptides of all three algorithms at 1% PSM-level FDR.

Since all algorithms have their inherent advantages and disadvantages, and different algorithms give different identification results, any single algorithm cannot capture all MS information. Implementing multiple algorithms can enhance the confidence of the peptide identification. The high-confidence peptides can estimate the quality of algorithm’s identification [11], [21]. We calculated the overlaps of the identified peptides for each two algorithms (Table S1). ‘High-confidence' peptides denote peptides found in at least two of the three search algorithms and calculated using the formula: 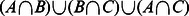, where A, B and C represent the identified peptides from Dispec, Mascot and Sequest. The number of high-confidence peptides identified by all the three algorithms was shown in Figure 5. In all cases, Dispec exceeded Mascot and Sequest in identifying high-confidence peptides, evidencing its quality to identify peptides. The detailed data are listed in Table S2.

### Summary

Here we presented a novel concept based on discriminability that takes three aspects of discriminability into consideration, and an open-source peptide scoring algorithm, Dispec, based on this concept. We validated the accuracy, robustness and compatibility of Dispec by comparing with two widely used algorithms, Mascot and Sequest. We believe that peptide matching discriminability information of each peak will be broadly accepted and integrated into new identification algorithms as a new native property of each MS peak, enhancing identification capacity and quality, which are essential for proteome studies.

## Supporting Information

Figure S1 **The selected peaks and the peptide matching number of each peak.**
(TIF)

Table S1 **Number of the same peptides identified between any two algorithms of Mascot, Sequest and Dispec.**
(XLS)

Table S2 **The high-confidence peptides of the three algorithms.**
(XLS)
